# Computer prediction of paratope on antithrombotic antibody 10B12 and epitope on platelet glycoprotein VI via molecular dynamics simulation

**DOI:** 10.1186/s12938-016-0272-0

**Published:** 2016-12-28

**Authors:** Wenping Liu, Guangjian Liu, Huiyun Zhou, Xiang Fang, Ying Fang, Jianhua Wu

**Affiliations:** 1 School of Bioscience and Bioengineering, South China University of Technology, Guangzhou, 510006 China; 2 Division of Birth Cohort Study, Guangzhou Women and Children’s Medical Center, Guangzhou Medical University, Guangzhou, 510623 China

**Keywords:** 10B12/GPVI interaction, Homology modeling, Molecular dynamics, Key residue analysis

## Abstract

**Background:**

Interaction between immunoglobulin-like receptor glycoprotein VI (GPVI) and collagen plays a central role in platelet activation and sequent firm adhesion. Of various antithrombotic agents targeting GPVI, antibody 10B12 is of great potential to block the GPVI-collagen interaction, but less is known about 10B12 paratope and GPVI epitope.

**Methods:**

Along the pathway in the computer strategy presented in our previous work, the 10B12/GPVI complex model was constructed through homology modeling and rigid-body docking, and the molecular dynamics (MD) simulation was used to detect the paratope residues on 10B12 and their partners on GPVI. Quantified by free and steered MD simulations, the stabilities of hydrogen bonds and salt bridges were used to rank the contributions of interface residues to binding of 10B12 and GPVI.

**Results:**

We predicted 12 key and seven dispensable residues in interaction of 10B12 to GPVI with present computational procedure. Besides of the 12 key residues, two are epitope residues (LYS^41^ and LYS^59^) which had been identified by previous mutation experiments, and others, including four epitope residues (ARG^38^, SER^44^, ARG^46^ and TYR^47^ on GPVI) and six paratope residues (GLU^1^, ASP^98^, GLU^102^, ASP^107^, ASP^108^ and ASP^111^ on 10B12), were newly found and also might be important for the 10B12–GPVI binding. The seven predicted dispensable residues on GPVI were had been illustrated in previous mutation experiments.

**Conclusions:**

The present computer strategy combining homology modeling, rigid body docking and MD simulation was illustrated to be effective in mapping paratope on antithrombotic antibody 10B12 to epitope on GPVI, and have large potential in drug discovery and antibody research.

## Background

Platelet-collagen interactions are believed to be of great significance in physiological hemostasis and pathological thrombosis [1]. During this process, binding of immunoglobulin (Ig)-like receptor glycoprotein VI (GPVI) to collagen is recognized as a central step [2], which leads to platelet integrin α_2_β_1_ activation and platelet granule release [3, 4]. Activated α_2_β_1_ could bind tightly to collagen and mediate firm adhesion of platelets to the injury site [5], while platelet granule contents would activate nearby circulating platelets and propagate thrombus formation. Therefore, GPVI becomes a primary therapeutic target for prevention of arterial thrombotic diseases such as heart attack and stroke [6].

Anti-GPVI antibody 10B12 is a potent antithrombotic agent and receives wide attention owing to the specific and efficient blocking of GPVI-collagen interaction [7, 8]. Injection of single-chain antibody fragment (scFv) 10B12 abolished binding of human platelets to human type III collagen and collagen-related peptide (CRP) [7, 8]. Mutagenesis experiments have been carried out to identify the epitope residues of GPVI interacting with 10B12 (Table 1). Smethurst et al. [7] found K59E substitution reduced binding of GPVI to CRP and 10B12, while ARG^60^, PHE^91^, ARG^117^, TYR^118^, PHE^120^, ARG^139^, SER^164^ and ARG^166^ were dispensable for the binding. O’connor et al. [9] proved that the mutation of the residue LYS^41^ to alanine abolished 10B12 binding while mutation of ARG^166^ had no significant effect. The messages for paratope on 10B12 and epitope on GPVI is not enough so that mapping paratope to epitope is required in increasing the efficacy of 10B12 [10, 11]. Traditional methods, including mutagenesis, phage display peptide libraries, X-ray crystallization and nuclear magnetic resonance, are usually expensive, time-consuming and blind [12]. Thus, computational methods such as rigid-body and flexible docking program are more and more used in paratope and epitope mapping [11, 13, 14]. Protein–protein interfaces, different from small molecules binding sites, are relatively large and discontinuous, leading to barriers in docking. And, protein flexibility also plays a great role in predicting druggable binding sites as well as the locations of interacting interfaces [11, 14–20]. The conformation transforming is missed completely in rigid body docking or partly in flexible docking, making it incapable to illustrate whether these residues are crucial or not for binding [12].

**Table 1:** Identified interaction residues between GPVI with 10B12 from mutagenesis experiments

Function	Name
Epitope residues on GPVI	LYS^59^, LYS^41^ [7, 9]
Dispensable residues on GPVI	ARG^60^, PHE^91^, ARG^117^, TYR^118^, PHE^120^, ARG^139^, SER^164^, ARG^166^ [7, 9]

Recently, a novel computational procedure to predict paratope residues and their partners via molecular dynamics (MD) simulations was developed and succeeded in mapping paratope to epitope of 6B4/GPIbα complex [21]. Along the pathway in the computer strategy described in our previous work [21], the 10B12/GPVI complex model was constructed through homology modeling and rigid-body docking, and MD simulation was used to detected the paratope residues on 10B12 and their partners on GPVI, with hypothesis that the dominant linkers between paratope and epitope are mainly contributed by the stable hydrogen bonds and salt bridges across the complex interface. Twelve key and seven dispensable residues in interaction of 10B12 to GPVI were predicted, in better agreement with previous mutation experiments. The results illustrated that **t**he present computer strategy might be effective in mapping paratope to epitope on GPVI/10B12, and have a wide application in guiding mutagenesis experiments and computer-aided antibody design.

## Methods

### Homology modeling

The structure of antibody 10B12 consists of a light chain (V_L_ and C_L_ domains), a heavy chain (V_H_ and C_H_ domains), and a 15-amino-acid (Gly_4_Ser)_3_ linker. The amino acid sequence of 10B12 was obtained from NCBI (Accession: AAN15184 and AAN15185). The structural templates of 10B12 were built up via PDB database search with BLAST for homology modeling [22]. The templates for heavy-, light-chains and (Gly4Ser)_3_ linker were the crystal structures of 8F9-Fab (PDB code 3F12, 83% identity), D5-Fab (PDB code 2CMR, 100% identity) and Fv antibody fragment (PDB code 1F3R, 100% identity), respectively. To yield the most likely VH–VL orientations, the crystal structure of anti-SARS ScFv antibody 80R (PDB code 2GHW) was used as a global template because it has the highest sequence identity (60%) with 10B12-ScFv among all antibody crystal structures containing the (Gly_4_Ser)_3_ linker in PDB based on the BLAST result. Meanwhile, both the light- and heavy-chain sequences of 10B12 were submitted to NCBI IgBlast (http://www.ncbi.nlm.nih.gov/igblast/) to identify complementarity determining regions (CDRs) of 10B12. CDR H1, H2, H3 in the heavy chain were made up of the sequences from 26th to 33rd residue, from 51st to 58th residue and from 96th to 112nd residue, whereas the sequences from 163rd to 168th residue, from 186th to 188th residue and from 225th to 233rd residue contributed to the CDR L1, L2 and L3 in light chain, respectively. 10B12 was first aligned to its templates by ClustalX [23], then the homology modeling was performed by Modeller 9v6 [24]. Eight 10B12 models of single chain Fv (ScFv) were built up and the model with a smallest Z-score value was regarded as a native-like model and selected for further docking to GPVI.

### Docking

The structure of unliganded GPVI (PDB code 2G17) was downloaded from PDB database. Docking of 10B12 to GPVI was performed with ZDOCK3.0 [25] and the shallow groove adjacent to the C’E loop of GPVI was designated as the binding site of 10B12 CDRs, as indicated in previous works [4]. In docking to a fixed 10B12, GPVI was translated and/or rotated with 6° sampling density in rotational space. Altogether 54,000 poses were generated with rigid-body docking and they were then analyzed and scored by Zrank [26]. Top 20 complexes ranking with negative Z-rank score were taken for visual inspection with the software visual molecular dynamics (VMD) [27]. The complex with the lowest Z-rank score was regarded as the best model [26], which was used in MD simulations.

### Free and steered MD simulations

Two software packages, VMD for visualization and modeling [27] and NAMD 2.9 program for MD simulations [28], were used here. The best 10B12/GPVI complex generated by ZDOCK was solvated with TIP3P water molecules in a rectangular box of 67.41 Å × 40.47 Å × 36.46 Å. Then Na^+^ and Cl^−^ ions were added to neutralize the systems at a 150 mM salt concentration. The CHARMM22 all-atom force field [29], along with cMAP correction for backbone, particle mesh Ewald algorithm for electrostatic interaction and a 12 Å cutoff for electrostatic and van der Waals interaction, were used to perform MD simulations with a periodical boundary condition and a time step of 2 fs.

First, the system was energy-minimized for 15,000 steps with all protein atoms fixed and for another 15,000 steps with all atoms free. Then the system was equilibrated for 60 ns twice with pressure and temperature control. The temperature was held at 310 K using Langevin dynamics, and the pressure was held at one atmosphere by the Langevin piston method. Time-curves of the temperature, total energy and root mean square deviation (RMSD) of heavy atoms were used to observe whether the system had been equilibrated. Two equilibrated complex structures were obtained from two corresponding equilibrated systems described above, and taken as the two initial conformations for free and steered MD simulations.

Free and steered MD simulations were run thrice on each equilibrated complex structure, respectively. Free MD simulation was conducted over 10 ns without control of temperature and pressure. In steered MD simulations, the C-terminal Cα atoms of both heavy and light chains of 10B12 were fixed and the C-terminal Cα atom of GPVI was steered with a virtual spring at a spring constant of 7000 pN/nm. A pulling over 9 ns was performed with time step 2 fs and a constant velocity of 1 nm/ns along the direction vertical to the line between two fixed C-terminal Cα atoms of 10B12.

All simulations were supported by National Supercomputer Center in Guangzhou.

### Survival ratio, normalized rupture time and stabilization index of a hydrogen bond

It was assumed that the interaction of a paratope residue to its partner can be scored out by the best stable one of H-bonds and/or salt bridges between this residue pair [21, 30]. A hydrogen bond was defined if the donor–acceptor distance and bonding angle were small than 3.5 Å and 30° respectively. But only the bond-length cutoff of 3.5 Å was applied to examine the salt bridges in binding site. The H-bonds and/or salt bridges across interface of complex were detected through VMD. Survival ratio of a H-bond (or salt bridge) was defined as the bond survival time in the period of simulation.

The survival ratio of the jth bond (ω_j_) expressed its thermal stability in free MD simulations. ω_j_ = max{ω_j1_, ω_j2_}, where ω_ji_ (i = 1, 2) is the mean survival ratio of the jth bond detected from thrice free MD simulations with the ith initial equilibrated complex conformation. The normalized rupture time α_j_ expressed the relative mechanical strength of the jth H-bond in steered MD simulations. α_j_ = θ_j_/max{θ_1_, θ_2_,…, θ_N_}, and θ_j_ = max{θ_j1_, θ_j2_}, where N is the total number of involved H-bonds, θ_ji_ is the mean rupture time of the jth bond detected from thrice steered MD simulations with the ith initial equilibrated complex conformation for i = 1, 2.

The stabilization index of the jth H-bond (HBSI_j_), which synthesizes the effects of both thermal stabilization and mechanical strength of the bond on the paratope–epitope interactions, was defined as that HBSI_j_ = (ω_j_ + α_j_)/2. We defined that a bond is low, moderate and high stable, if anyone of its survival ratio, normalized rupture time and HBSI index lies in the region from 0 to 0.3, from 0.3 to 0.55 and from 0.55 to 1.0, respectively.

## Results

### The best docking model provided less information of epitope and paratope residues of 10B12/GPVI

**Fig. 1:**
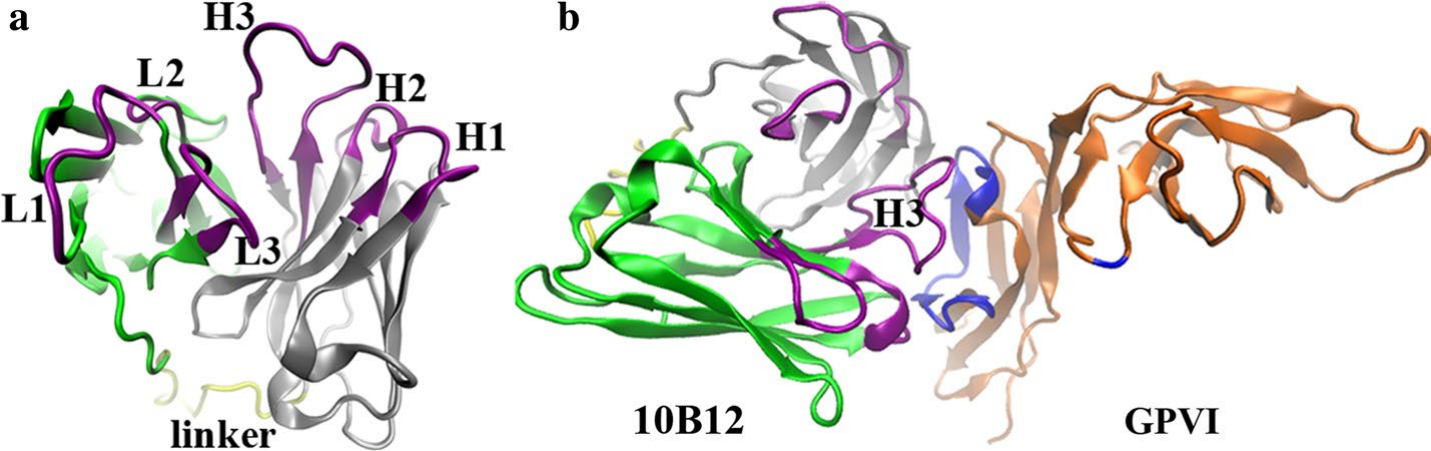
The best docking model of 10B12 in binding without or with GPVI. **a** The best model of 10B12 from Homology modeling, including the light chain (*cyan*), heavy chain (*gray*), linker (*yellow*) and the six CDR regions (*purple*). **b** Conformation of 10B12 (*left*) bound to GPVI (*right*, *orange*). The binding sites (*blue*) on GPVI to 10B12 were shown here

We obtained a model of 10B12-ScFv through homology modeling (see “Methods” section). Its six CDRs, which located on the top of both light- and heavy-chains, mainly contribute to binding of 10B12 to GPVI (Fig. 1a). After that, 54,000 poses were generated by rigid-body docking of the above 10B12-ScFv to ligand-free GPVI (see “Methods” section). The 2718th complex (Fig. 1b) with the lowest Z-rank score of −106.48 was picked out. This complex model may be the best one, because the lowest Z-rank score means its conformation is most energy favorable.

Subsequent conformational analysis with VMD revealed that the selected 10B12/GPVI model might be meaningful biologically. Katsunori Horii et al. [4, 9] found that a shallow groove formed by basic residues on the surface of D1 domain surface including LYS^41^, LYS^59^, ARG^60^ and ARG^166^, adjacent to the C’E loop, was the main binding site on GPVI to collagen. From Fig. 1b, we could clearly see that this region (blue) was mostly occupied by CDR H3 of 10B12 (purple), suggesting a competitive binding of 10B12 and collagen to GPVI.

Furthermore, we analyzed the H-bonds and salt bridges at the interface of the 2718th complex with VMD to get the information about epitope and paratope residues [21, 30]. Mutation data demonstrated that both LYS^59^ and LYS^41^ were key residues (Table 1) [7, 9]. We found from 2718th complex that there existed three H-bonds and one salt bridge, involving four residues (GLU^21^, TYR^47^, GLN^50^ and LYS^59^) on GPVI and four residues (GLU^1^, GLY^26^, ASP^107^ and LYS^186^) on 10B12 (Table 2). From these eight residues, just LYS^59^ emerged and LYS^41^ did not. It means that, docking analysis of one static complex only provided less information on intermolecular interaction at binding site even if this conformation is energy favorable, as shown in our previous research [21]. The reason might be that the molecular recognition and drug binding are dynamic processes [31] and 10B12/GPVI complex in water should undergo a random conformational transition.

**Table 2:** Residue interactions between 10B12 and GPVI from docking model

No.	Hydrogen bond		Salt bridge	
	GPVI	10B12	GPVI	10B12
1	GLN^50^	GLU^1^	GLU^21^	LYS^186^
2	TYR^47^	GLY^26^		
3	LYS^59^	ASP^107^		

### Thermal stabilization of H-bonds marked paratope and epitope residues of 10B12/GPVI complex

**Fig. 2:**
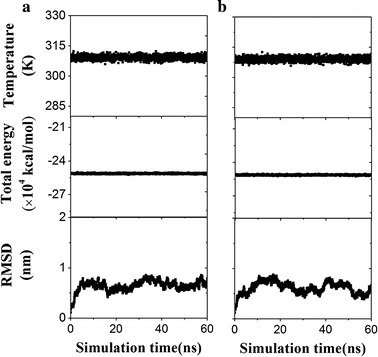
Variation of the temperature, total energy and RMSD of heavy atoms of GPVI/10B12 complex versus simulation time. **a**, **b** Denote two independent system equilibrium processes (**a**, **b**) of GPVI/10B12 complex, respectively. Each system equilibrium process was demonstrated by the time-courses of the temperature, total energy and RMSD of heavy atoms of the complex

It was demonstrated that paratopes and epitopes could be mapped by H-bonds with higher thermal stabilization in free MD simulations [21]. Here, the 2718th complex, the best model of 10B12/GPVI, was chosen to perform MD simulations. System equilibrium was performed twice along a same protocol of energy minimization. From the time-curves of the temperature, total energy and RMSD of heavy atoms (Fig. 2), it was shown that the two systems were equilibrated after 30 ns. We examined the events of breaking and forming of bonds by performing free MD simulations thrice on each of initial equilibrated conformation I and II of 10B12/GPVI complex for 10 ns. Survival ratios of all detected H-bonds were listed in Table 3, which indicated that, in comparison with docking results (Table 2), two H-bonds and one salt bridge were lost but 24 H-bonds were newly detected. Of these detected bonding, the 2nd bond only appeared in the simulations for the equilibrated conformation I while the 5th–8th bonds only emerged from the simulations for the equilibrated conformation II (Table 3). The characteristic of initial state-dependent H-bonds formation suggests that a number of simulations in parallel are required to better understand the residue interactions across protein interface [21].

**Table 3:** Summary of survival ratios, rupture time and involved residues of hydrogen bonds detected from free and steered MD simulations

Bond	GPVI		10B12		Survival ratio			Rupture time		
No.	Residue	Atom	Residue	Atom	I	II	ω	I (ns)	II (ns)	α
1	ARG^46^	NH2	ASP^98^	OD1	0.39 ± 0.04	0.94 ± 0.01	0.94	1.20 ± 0.31	2.18 ± 0.81	0.75
2	LYS^41^	NZ	GLU^102^	OE2	0.79 ± 0.08		0.79	2.88 ± 1.08	0.14 ± 0.11	1.00
3	ARG^38^	NH2	GLU^1^	OE1	0.42 ± 0.30	0.66 ± 0.14	0.66	0.69 ± 0.80	1.71 ± 0.50	0.59
4	TYR^47^	OH	GLU^1^	OE2	0.09 ± 0.04	0.65 ± 0.15	0.65		1.48 ± 0.23	0.51
5	ARG^166^	NH2	GLU^102^	OE1		0.56 ± 0.04	0.56		0.44 ± 0.23	0.15
6	ARG^46^	NH2	ASP^111^	OD2		0.54 ± 0.29	0.54		2.41 ± 0.71	0.83
7	ARG^166^	NH1	GLU^102^	OE2		0.54 ± 0.06	0.54		0.36 ± 0.29	0.12
8	LYS^59^	NZ	ASP^107^	OD2		0.53 ± 0.27	0.53		0.44 ± 0.25	0.15
9	ARG^46^	NH1	ASP^98^	OD1	0.50 ± 0.13		0.50	2.48 ± 0.53		0.86
10	LYS^59^	NZ	ASP^108^	OD1	0.33 ± 0.10	0.48 ± 0.38	0.48	0.56 ± 0.30	0.30 ± 0.27	0.19
11	SER^44^	OG	GLU^102^	OE2	0.45 ± 0.11		0.45	1.28 ± 0.98	0.05 ± 0.09	0.44
12	ARG^38^	NH1	GLU^1^	OE2	0.44 ± 0.21	0.45 ± 0.09	0.45	0.72 ± 0.80	1.50 ± 0.49	0.52
13	ARG^46^	NE	GLU^102^	OE1	0.40 ± 0.08		0.40	1.54 ± 0.10	0.02 ± 0.04	0.53
14	SER^44^	OG	GLU^102^	OE1	0.40 ± 0.10		0.40	1.52 ± 0.35	0.01 ± 0.01	0.53
15	LYS^59^	NZ	ASP^108^	OD2	0.39 ± 0.08	0.36 ± 0.40	0.39	0.44 ± 0.31		0.15
16	ARG^166^	NH2	GLU^102^	OE2		0.30 ± 0.01	0.30		0.61 ± 0.24	0.21
17	LYS^59^	NZ	ASP^107^	OD1		0.29 ± 0.30	0.29			
18	ARG^46^	NH2	ASP^98^	OD2	0.27 ± 0.03	.	0.27	1.18 ± 0.92		0.41
19	ARG^38^	NH2	GLU^1^	OE2	0.25 ± 0.17	0.21 ± 0.16	0.25	0.56 ± 0.71	0.07 ± 0.07	0.19
20	ARG^166^	NH1	GLU^102^	OE1		0.23 ± 0.02	0.23		0.63 ± 0.27	0.22
21	ARG^46^	NH1	ASP^111^	OD2		0.23 ± 0.13	0.23		0.71 ± 0.35	0.24
22	SER^43^	OG	GLU^102^	OE1	0.22 ± 0.20		0.22			
23	TYR^32^	OH	ASP^167^	OD2	0.17 ± 0.35		0.17			
24	ARG^38^	NH1	GLU^1^	OE1	0.11 ± 0.08	0.12 ± 0.10	0.12	0.57 ± 0.71		0.20
25	TYR^47^	OH	GLU^1^	OE1	0.01 ± 0.01	0.09 ± 0.12	0.09		0.09 ± 0.08	0.03

The heading I and II denote two different equilibrated complex conformation of 10B12 bound to GPVI, and the values (Column 8 and 11) of express the thermal and mechanical stabilities of the bonds detected from free and steered MD simulations thrice with two different equilibrated conformations (see “Methods” section). The superscript numbers on residues (Column 2 and 4) designate the positions of their respective involved residues in sequences of GPVI and 10b12 with serial numbering, the donor- and acceptor-atoms (Column 5) on paratope residues (Column 4) together with their respectively partners (Column 3) on epitope residues (Column 2) contribute to bonds in binding site. All bonds, which were derived from thrice independent free and steered MD simulations with equilibrated conformation I and II, respectively, were designated by nonzero values (mean ± SD) of survival ratios and rupture times of bonds

**Fig. 3:**
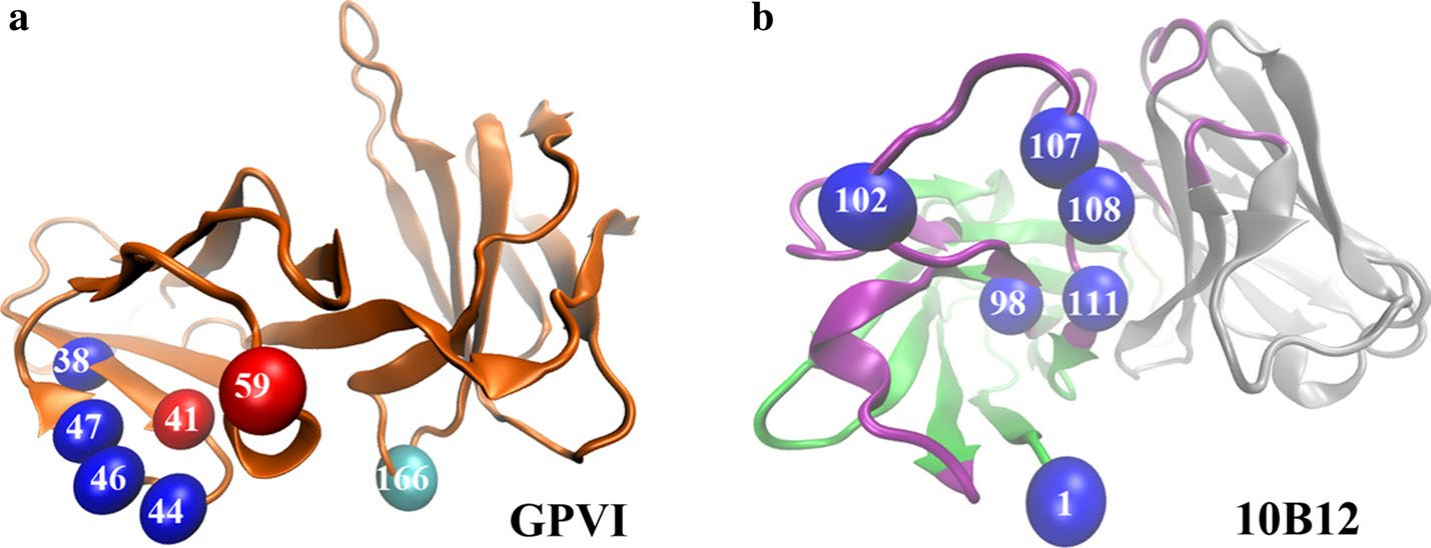
The predicted interaction residues on interface of GPVI-10B12. **a** The key residues on GPVI and **b** the key residues on 10B12. *Red* stands for the identified residues via mutagenesis experiments, and *blue* stands for the key residues which were predicted by MD simulation without mutagenesis data, *cyan* stands for the dispensable residues demonstrated by mutation data. All interaction residues on interface of GPVI-10B12 complex were predicted here by mean survival ratio, normalized mean rupture time or HBSI index

The maximum value of mean survival ratios with initial equilibrated conformation I and II was taken as the mean survival ratio (ω) of a H-bond (see “Methods” section, Table 3). Twenty-five H-bonds were clustered into three groups of low, moderate and high thermal stabilization by their corresponding mean survival ratio values ranging from 0 to 0.3, from 0.3 to 0.55 and from 0.55 to 1.0, respectively. The H-bonds with moderate and high thermal stabilization were used to map paratope to epitope residues for preventing loss of potential key residues. Involved in 16 H-bonds from the 1st to the 16th bonds in Table 3), 13 residues, including ARG^38^, LYS^41^, SER^44^, ARG^46^, TYR^47^, LYS^59^ and ARG^166^ on GPVI as well as GLU^1^, ASP^98^, GLU^102^, ASP^107^, ASP^108^ and ASP^111^ on 10B12 (Fig. 3), were sorted out with a cutoff of 0.3. As expected, the key residue LYS^41^, which was identified to be dominant for the binding by the mutagenesis experiment [9] but not observed in docking analysis, formed the high stable 2nd H-bond with GLU^102^ on 10B12. The key residue LYS^59^ involved in three H-bonds (the 8th, the 10th and the 15th bonds), explaining why its mutation seriously impaired the binding between GPVI and 10B12 [7]. Out of expect, ARG^166^ formed three H-bonds (the 5th, 7th and 16th bonds) with GLU^102^ but it was proved dispensable for the binding by the experiment [9], possibly coming from that this mutation might enhance the interactions of GLU^102^ to its two other partners LYS^41^ and SER^44^ on GPVI (Table 3). Taken together, two critical residues on GPVI (LYS^41^ and LYS^59^) were both sorted out by H-bonds with moderate and high thermal stabilization and the simulations also predicted ten potential key residues without mutagenesis experiments data.

### Mechanical H-bonding stabilization is another mark for key interaction residues across 10B12/GPVI complex interface

Similar to the thermal stabilization reflected by the survival ratios of H-bonds, the mechanical stabilizations of H-bonds was also used to map paratope and epitope residues [21]. Rupture times of all H-bonds (Table 3) were detected from steered MD simulations which were performed thrice for each of the equilibrated conformation I and II (see “Methods” section). The random feature and initial-state dependence of H-bonds were also observed, similar to those in the free MD simulations. For example, the 5th–8th bonds only appeared in the simulations for the equilibrated conformation II, while and 9th bond emerged only in the simulations for the equilibrated conformation I (Table 3). Normalized mean rupture time (α) of each H-bond was calculated and listed in Table 3 (see “Methods” section). Seven H-bonds, including 1–4th, 6th, 9th, 11–14th and 18th bonds, had high mechanical stabilization for their normalized mean rupture time α larger than 0.3. With these H-bonds, nine residues were predicted to be paratope and epitope residues, including five (ARG^38^, LYS^41^, SER^44^, ARG^46^, TYR^47^) on GPVI and four (GLU^1^, ASP^98^, GLU^102^, ASP^111^) on 10B12 (Table 3; Fig. 3).

A bonding may be stable thermally rather than mechanically and vice versa. It was shown from Table 3 that, the 5th, the 7th and the 8th bonds had higher thermal stabilizations for their survival ratio larger than 0.5, but their mechanical stabilizations were low because of the normalized mean rupture time α smaller than 0.2; the 18th bond was stable mechanically rather than thermally for its mean survival ratio of 0.27 and its normalized mean rupture time α of 0.41. Of the above nine residues proposed through mechanical stabilization, LYS^41^ had been identified as an epitope residue by mutagenesis experiments, and others had not. In comparison with key residues from free MD simulations, four residues (LYS^59^, ARG^166^ on GPVI and ASP^107^, ASP^108^ on 10B12) were missed in the steered MD simulations, where mutation data demonstrated LYS^59^ dominant and ARG^166^ dispensable for binding [9].

### Mapping paratope to epitope via H-bond stabilization index

To score synthetically the thermal and mechanical stabilization of a hydrogen bonding, hydrogen bond stabilization index (HBSI), which was presented in our previous work for antithrombotic antibody 6B4 [21], were calculated by the average of the mean survival ratio and the normalized mean rupture time of each of all detected bond (see “Methods” section). All H-bonds were clustered into three groups with low, moderate and high stabilization by HBSI values ranging from 0 to 0.3, 0.3 to 0.55 and 0.55 to 1.0, respectively.

The H-bonds with HBSI values (from 0.33 to 0.89) ranked in top 15 were predicted to be stable (Table 4). Thirteen residues, seven (ARG^38^, LYS^41^, SER^44^, ARG^46^, TYR^47^, LYS^59^, and ARG^166^) on GPVI and six (GLU^1^, ASP^98^, GLU^102^, ASP^107^, ASP^108^, ASP^111^) on 10B12 and two pre-identified epitope residues (LYS^41^ and LYS^59^), were involved in above stable bonds. Of these predicted key residue, LYS^41^ and LYS^59^ are the identified epitope residues (Fig. 3) [7, 9]. The key residues from HBSI analysis were the same as those from free MD simulations, but the stabilization ranking of their involved H-bonds were different. The bond ranked 2nd in survival ratio but 1st in the normalized mean rupture time due to the highest mechanical stabilization (Table 4), highlighting the importance of epitope residue LYS^41^; and, the 18th bond was missed from free MD simulations but ranked 14th in HBSI, benefiting from its high mechanical stabilization too. It indicated that, mechanical stabilization of bonding should be important in predicting paratope–epitope interactions via MD simulation, and HBSI might include more detailed information about paratope–epitope interactions in comparison with survival ratio and rupture time.

**Table 4:** The stable H-bonds with HBSI values >0.3

Rank	Bond no.^a^	HBSI	Interaction residue pairs	
			GPVI	10B12
1	2	0.89	LYS41	GLU102
2	1	0.85	ARG46	ASP98
3	6	0.69	ARG46	ASP111
4	9	0.68	ARG46	ASP98
5	3	0.63	ARG38	GLU1
6	4	0.58	TYR47	GLU1
7	12	0.48	ARG38	GLU1
8	13	0.47	ARG46	GLU102
9	14	0.46	SER44	GLU102
10	11	0.45	SER44	GLU102
11	5	0.36	ARG166	GLU102
12	8	0.34	LYS59	ASP107
13	10	0.34	LYS59	ASP108
14	18	0.34	ARG46	ASP98
15	7	0.33	ARG166	GLU102

^a^The bond no in column 2 were same as those in Table 3

## Discussion

Mapping paratope to epitope residues of potent antithrombotic antibody 10B12 is an essential step to re-design this antibody for higher affinity or other desired modifications. However, only two epitope residues without their paratope residues were identified through mutation experiments [7, 9]. With a novel computational procedure proposed in our previous work for the antithrombotic antibody 6B4 [21], we here investigated the paratope and epitope residues of 10B12/GPVI complex. Successfully, the two known identified epitope residues (LYS^41^ and LYS^59^) on GPVI were predicted as critical residues, and seven residues (ARG^60^, PHE^91^, ARG^117^, TYR^118^, PHE^120^, ARG^139^, SER^164^ and ARG^166^), being dispensable for the binding [7], did not listed in the key interaction residues through our computational procedure. These results suggested that our computational procedure had higher sensitivity and specificity in mapping paratope to epitope of the 10B12/GPVI complex. And, other newly predicted ten residues, four (ARG^38^, SER^44^, ARG^46^, and TYR^47^) on GPVI and six (GLU^1^, ASP^98^, GLU^102^, ASP^107^, ASP^108^ and ASP^111^) on 10B12, might be important for binding. The reason for this success lies in that MD simulations can mimic the atom fluctuations and conformational changes of biomolecules in liquid or physiological environment [32, 33].

As illustrated in our previous work for the antithrombotic antibody 6B4 [21], the present results indicated again that as a solid linker between a paratope residue and its partner, the strong hydrogen bonding across complex interface was a better mark in mapping paratope to epitope; the key and abundant messages of paratope and epitope residues could be extracted from MD simulation rather than docking analysis, because MD simulation would build up a quasi-complete complex conformation space, from which most of hydrogen bonding could be detected; the weak thermal disturbing did not cross the barrier of conformational transform but mechanical stretch did, this was why some key interaction residues had not emerged from free MD simulation but could be detected from steered MD simulation; and in the present computer strategy, three different complex structures on equilibrium state, at least, should be used to the subsequent MD simulation, because of that, the simulated evolution of complex conformation was dependent on the initial structure of the complex.

However, it should be noted that the residue ARG^166^ on GPVI was a false positive in our study. Maybe, the reason might come from that the simulation times were not long enough and the sampling spaces were not large enough. It remains a room for improvement in the accuracy of the procedure such as through extending the simulation time or selecting more initial conformations.

## Conclusions

The present computer strategy, combining homology modeling, rigid body docking and MD simulation, should be effective not only in mapping paratope on antithrombotic antibody 10B12 to epitope on GPVI but also in other antibody researches, have large potential in drug discovery and antibody research, provide useful clues to mutation experiments and assist the computer-aided antibody design.
